# Bacterial communities of hookah tobacco products are diverse and differ across brands and flavors

**DOI:** 10.1007/s00253-022-12079-7

**Published:** 2022-08-05

**Authors:** Leena Malayil, Suhana Chattopadhyay, Emmanuel F. Mongodin, Amy R. Sapkota

**Affiliations:** 1 Maryland Institute for Applied Environmental Health, University of Maryland School of Public Health, College Park, MD USA; 2 Institute for Genome Sciences, University of Maryland School of Medicine, Baltimore, MD USA; 3 Present Address: Division of Lung Diseases, National Heart, Lung and Blood Institute (NHLBI), National Institutes of Health (NIH), Bethesda, MD USA

**Keywords:** Tobacco, Hookah, Shisha, Microbiome, Bacteria

## Abstract

**Abstract:**

Young adults are increasingly using non-cigarette products, such as hookahs, since they are perceived as healthier alternatives to cigarette smoking. However, hookah users are exposed to not only carcinogenic compounds but also microorganisms that may play an active role in the development of both infectious and chronic diseases among users. Nevertheless, existing hookah research in this area has focused only on microorganisms that may be transferred to users through the smoking apparatus and not on bacterial communities associated with hookah tobacco. To address this knowledge gap, we conducted time-series experiments on commercially available hookah brands (Al Fakher (flavors: two apple, mint, and watermelon) and Fumari (flavors: white gummy bear, ambrosia, and mint chocolate chill)) stored under three different temperature and relative humidity conditions over 14 days. To characterize bacterial communities, the total DNA was extracted on days 0, 5, 9, and 14, PCR-amplified for the V3V4 region of the bacterial 16S rRNA gene, sequenced on the Illumina HiSeq platform, and analyzed using R. Diversity (alpha and beta) analyses revealed that the microbiotas of Fumari and Al Fakher products differed significantly and that flavor had a significant effect on the hookah microbiota. Overall, *Pseudomonas*, *Bacillus*, *Sphingomonas*, and *Methylobacterium* were the predominant bacterial taxa across all products*.* Additionally, we observed compositional differences between hookah brands across the 14-day incubation*.* These data suggest that the bacterial communities of hookah tobacco are diverse and differ across brands and flavors, which may have critical implications regarding exposures to specific bacteria among hookah users.

**Key points:**

• *Commercial hookah products harbor diverse bacterial communities*.

• *Brands and flavors impact the diversity of these communities*.

• *Research on their viability and transmission to users’ respiratory tracts is needed*.

**Graphical abstract:**

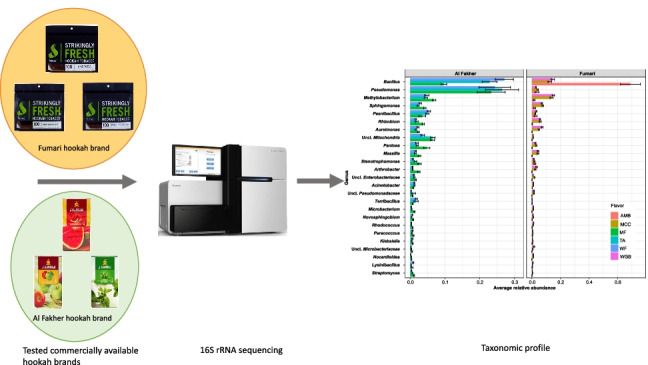

**Supplementary Information:**

The online version contains supplementary material available at 10.1007/s00253-022-12079-7.

## Introduction

Smoking tobacco through water pipes, also known as hookah, shisha, nargileh, argileh, hubble-bubble, and goza (depending on the country of origin), has been taking place for centuries as a part of the cultural traditions of a variety of regions, including the Eastern Mediterranean and Middle East, as well as parts of Asia (Rice 2012). However, hookah use has gained increasing popularity in the USA among children and young adults especially women over the past two decades (Jordan and Delnevo 2010; Smith et al. 2011). A survey-based study investigated hookah usage among 6th–12th grade students (*n* = 20,675) in the USA and reported that 10.5% smoke hookah (Agaku et al. 2018). Additionally, Roberts et al. (2017) observed a higher prevalence of hookah use in urban areas compared to rural areas (Roberts et al. 2017). Hookah smoking is widely popular among youth and women for two main reasons: (1) the misperception that health risks associated with hookah smoking are lower than those associated with other forms of tobacco smoking and (2) the widespread availability of hookah flavors that appeal to youth and women (Palamar et al. 2014; Dadipoor et al. 2019).

Hookah smokers use a special apparatus that has a head, a metal body, a water bowl, and a flexible hose with a mouthpiece. The smokers burn charcoal on top of a tobacco preparation, which is a mixture of tobacco, glycerin, water, and flavorings. The resulting smoke then bubbles through the water bowl before users inhale it via the mouthpiece. Since the smoke passes through water before being inhaled, many users hold the incorrect perception that smoking through a water pipe is less harmful compared to cigarette or cigar smoking (Kandela 1997). While the negative effects of tobacco smoking on one’s health are well known and include cancer and pulmonary and cardiovascular diseases, the health consequences associated with hookah smoking, including exposures to harmful toxins and spreading infectious diseases through pipe sharing, are understudied. Hookah use has been associated with chronic bronchitis, oral cancer, cardiovascular diseases, and infectious diseases (Blachman-Braun et al. 2014). A comparative meta-analysis of the lung function of cigarette smokers, hookah smokers, and nonsmokers revealed no significant difference in spirometric tests among cigarette smokers and hookah smokers (Raad et al. 2011). However, two other studies revealed that the frequency of chronic bronchitis is higher in hookah smokers compared to cigarette smokers (Mutairi et al. 2006; Mohammad et al. 2008). A cohort study inclusive of 36 hookah users and 36 control subjects showed that hookah usage is an important etiologic factor for oral cancer and dysplastic lesions (Taghibakhsh et al. 2019).

Mainstream hookah smoke contains several toxicants, including nicotine, carcinogenic polycyclic aromatic hydrocarbons, and heavy metals (Qasim et al. 2019). Recently, the presence of bacterial lipopolysaccharide (LPS) and fungal biomass was also identified in hookah mainstream smoke (Markowicz et al. 2014). Multiple studies have also shown an association between hookah smoking and infectious diseases, attributed to sharing of water pipes among users (Martinasek et al. 2018; Akl et al. 2010; Szyper-Kravitz et al. 2001; Munckhof et al. 2003). For example, Martinasek et al. (2018) observed the highest bacterial prevalence and diversity in the mouthpiece of the waterpipe (Martinasek et al. 2018). Based on data from 10 hookah bars, this study not only identified both Gram-positive and Gram-negative bacteria but also antibiotic-resistant bacteria from the mouthpiece of the sampled water pipes (Martinasek et al. 2018). *Mycobacterium tuberculosis* (Akl et al. 2010), *Aspergillus* (Szyper-Kravitz et al. 2001), and other spore-producing fungi (Moustafa and Abdelzaher 2015) also have been isolated from water pipes and their potential for transmission to users has been demonstrated. For example, pulmonary tuberculosis was identified among a cluster of young Caucasian hookah users in Queensland, Australia (Munckhof et al. 2003). Other studies have also linked hookah usage to the transmission of *Helicobacter pylori* (El Barrawy et al. 1997) and *Aspergillus* spores (Szyper-Kravitz et al. 2001). InterestinglyHabib et al. (2001)reported that the hookah smoking community might be prone to hepatitis C virus infections due to the sharing of mouthpieces (Habib et al. 2001). Similarly, a recent study by Hani et al. (2018) observed the presence of 40 bacterial genera among three investigated water pipes (Hani et al. 2018). In 2019, Alagaili et al. reported that hookah smokers were at a high risk for transmission of Middle East Respiratory Syndrome (MERS-CoV) (Alagaili et al. 2019). With the current SARS-CoV-2 pandemic, smoking has emerged as an independent risk factor not only for the transmission of but also for the severity of COVID-19 (Liu et al. 2020; Shekhar and Hannah-Shmouni 2020). All of these studies indicate that microbial contamination of hookah apparatus may be a mode of transmission for infectious diseases.

Nevertheless, existing studies on the negative health effects associated with hookahs have mostly focused on microbial loads in the various physical components of the water pipe. However, the microbial communities that may be present in the hookah tobacco itself have not been fully characterized, even though multiple studies have identified and characterized a plethora of microorganisms in other tobacco products (e.g., cigarettes, little cigars, cigarillos, and smokeless tobacco), including *Actinomycetes*, *Acinetobacter*, *Bacillus*, *Burkholderia*, *Clostridium*, *Klebsiella*, *Pseudomonas*, *Serratia*, *Campylobacter*, *Enterococcus*, *Proteus*, and *Staphylococcus* (Sapkota et al. 2009; Tyx et al. 2016; Han et al. 2016; Chopyk et al. 2017a, 2017b; Smyth et al. 2017, 2019; Chattopadhyay et al. 2019; Malayil et al. 2020). To address this knowledge gap, we performed a time series experiment to characterize the bacterial communities present in hookah tobacco from two top brands: Al Fakher and Fumari. In addition, we investigated three commonly used flavors from each brand to examine how flavors might impact the hookah tobacco bacterial communities, which may potentially affect users’ health.

## Methods

### Sample collection

We characterized six commercially available hookah tobacco products: three flavors of Al Fakher (two apple [TA], mint [MF], and watermelon [WF]) and three flavors of Fumari (white gummy bear [WGB], ambrosia [AMB], and mint chocolate chill [MCC]). All products were purchased online and shipped to the University of Maryland, College Park, MD, USA. Three lots of each of the six hookah products were incubated in the laboratory for 14 days under three different experimental conditions to simulate regular user storage conditions: room (20 °C and 50% relative humidity), refrigerator (5 °C and 18% relative humidity), and pocket (25 °C and 30% relative humidity). Subsamples were collected and tested in replicate on days 0, 5, 9, and 14. A total of 432 samples were tested over the course of the study.

### DNA extraction, 16S rRNA gene PCR amplification, and sequencing

Total DNA was extracted under sterile laboratory conditions from 0.2 g of all hookah tobacco samples using previously published methods (Chopyk et al. 2017a, 2017b; Chattopadhyay et al. 2019; Smyth et al. 2019; Malayil et al. 2020). Additionally, negative extraction controls were included at every step of sample processing to ensure no exogenous DNA contaminated the samples. With the extracted DNA, the V3V4 hypervariable region of the 16S rRNA gene was then amplified using the universal primers 319F (ACTCCTACGGGAGGCAGCAG) and 806R (GGACTACHVGGGTWTCTAAT (Fadrosh et al. 2014). The PCR reaction and conditions have been extensively described in previously published articles (Chopyk et al. 2017a, 2017b; Chattopadhyay et al. 2019; Holm et al. 2019; Smyth et al. 2019; Malayil et al. 2020). Amplicon presence was confirmed using gel electrophoresis, and amplicons were cleaned up and normalized using the SequelPrep Normalization Kit (Invitrogen Inc., Carlsbad, CA, USA) prior to pooling and sequencing.

### Sequencing quality filtering and data analysis

After sequencing, 16S rRNA paired-end read pairs were assembled using PANDAseq (Masella et al. 2012), de-multiplexed, and trimmed of artificial barcodes and primers. They were then assessed for chimeras using UCHIME in de novo mode implemented in Quantitative Insights Into Microbial Ecology (QIIME; release v.1.9.1) (Caporaso et al. 2010). Quality trimmed sequences were then clustered de novo into operational taxonomic units (OTUs) at a 97% confidence threshold, and taxonomic assignments were assigned using the GreenGenes database (DeSantis et al. 2006) through VSEARCH (Rognes et al. 2016). The following packages in RStudio (v.1.1.423) were used for downstream data analysis and visualization: biomformat (v.1.2.0) (McMurdie PJ and Paulson JN 2017), vegan (v.2.4.5) (Oksanen et al. 2017), ggplot2 (v.3.1.0) (Wickham 2009), phyloseq (v.1.19.1) (McMurdie and Holmes 2013), Bioconductor (v.2.34.0) (Huber et al. 2015), and metagenomeSeq (v.1.16.0) (Paulson et al. 2013).

To address uneven sampling depth, beta diversity normalization of reads was completed using metagenomeSeq’s cumulative sum scaling (CSS) (Paulson et al. 2013). Beta diversity was computed using the Bray–Curtis dissimilarity index, and statistical analysis was calculated using Analysis of similarities (ANOSIM; 999 permutations) on normalized data. DESeq2 (v.1.14.1) (Love et al. 2014) was utilized to compute statistically significantly different (*p* < 0.05) OTUs between brands (Fumari and Al Fakher) at an alpha of 0.05 (on OTUs that were at > 0.1% relative abundance). Network analyses were also completed (for OTUs with a maximum relative abundance > 5% in at least one sample) to discern shared and unique OTUs across brands and flavors. These analyses were completed using several R packages: vegan (v.2.4.5) (Oksanen et al. 2017), dplyr (v.0.7.8) (Wickham et al. 2018), circlize (v.0.4.5) (Gu et al. 2014), reshape2 (v.1.4.3) (Wickham 2007), and stringr (v.1.3.1). The network analysis plots were visualized using Cytoscape (v.3.7.2).

## Results

### Sequencing dataset

A total of 432 samples were successfully PCR amplified and sequenced, generating a total of 16,402,317 sequences across all samples and 4,565 operational taxonomic units (OTUs). Across the samples that were successfully PCR-amplified and sequenced, the minimum number of reads was 20, and the maximum was 141,489, with an average number of sequences per sample of 37,968.33 (+ / − 30,858.61 SD). Despite several rounds of troubleshooting PCR amplification and sequencing of Fumari ambrosia samples, these samples could only be sequenced at a significantly lower sequencing depth compared to other samples (Supplemental Fig S1).

To ensure that all samples in the final dataset were sequenced to an appropriate coverage level across study groups, the Good’s estimate of coverage was calculated, and samples with Good’s value < 0.85 were removed. These included 6 Al Fakher two apple samples, 1 Al Fakher watermelon sample, 16 Fumari ambrosia samples, 1 Fumari mint chocolate chill sample, and 1 Fumari white gummi bear sample (Supplemental Fig S2). After filtering of *Cyanobacteria* sequences (sequences likely amplified from plant chloroplast DNA) and pruning of low abundance taxa (OTUs with less than 10 sequences), the final dataset analyzed contained 4,499,475 sequences clustered into 2,972 OTUs from 407 samples.

### Microbiota differences between brands and flavors at baseline

For the Al Fakher brand, we observed that the mint flavor (MF) had a significantly (*p* < 0.0001) higher alpha diversity for both metrics (observed: 299.72 + / − 81.01; Shannon: 4.25 + / − 0.43) when compared to the two apple (TA) flavor (observed: 184.39 + / − 49.17; Shannon: 3.60 + / − 0.99) and a significantly higher alpha diversity regarding only the observed metric compared to the watermelon flavor (WA) (195.94 + / − 67.59). For the Fumari brand, tobacco-associated bacteria in the ambrosia flavor (AMB) were characterized by significantly (*p* < 0.0001) lower alpha diversity (observed: 43.11 + / − 32.93; Shannon: 2.15 + / − 0.76) when compared to the other two Fumari flavors (mint chocolate chill [MCC] (observed: 310.56 + / − 82.77; Shannon: 4.27 + / − 0.38) and white gummy bear [WGB] (observed: 273.89 + / − 97.29; Shannon: 4.14 + / − 0.46)) across both alpha diversity metrics. These alpha-diversity results indicate that flavor significantly affects hookah-associated bacterial communities (Supplemental Fig. S3).

**Fig. 1:**
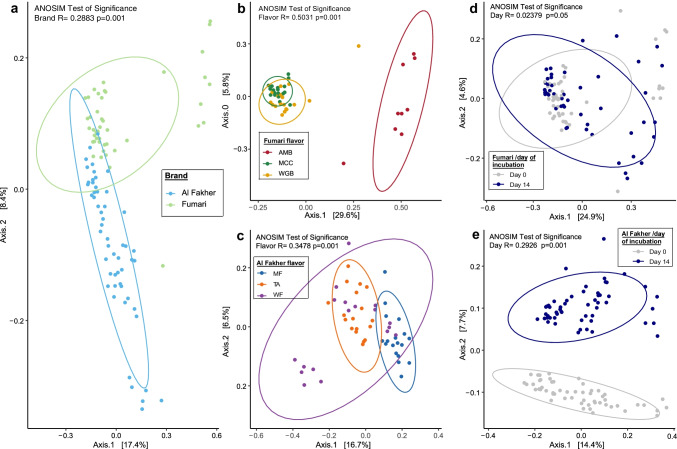
Principal coordinate analysis plots of Bray–Curtis dissimilarity distances. Comparison between **a** Al Fakher (light blue) and Fumari (light green) brands; **b** Fumari flavors: red, ambrosia (AMB); green, mint chocolate chill (MCC); and brown, white gummy bear (WGB); **c** Al Fakher flavors: dark blue, mint (MF); orange, two apple (TA); and purple, watermelon (WF) at baseline; **d** day 0 (gray) and day 14 (blue) in Fumari brand; **e** day 0 (gray) and day 14 (blue) in Al Fakher brand. Solid ellipses represent the 95% confidence intervals for brands (a), flavors (b and c), and days of incubation (d and e)

Beta diversity analyses also indicated that the hookah bacterial microbiota is influenced by both brand and flavor. Bacterial community structures were significantly different between the two brands (ANOSIM R: 0.2883, *p* = 0.001) (Fig. 1a). Additionally, the comparison between the three flavors among the individual brands (Al Fakher and Fumari) also demonstrated significant differences in beta diversity (Fig. 1b and c). Overall, the diversity measures indicate that the bacterial microbiotas in hookah tobacco products are highly dependent on the tobacco brand and are significantly influenced by flavoring.

**Fig. 2:**
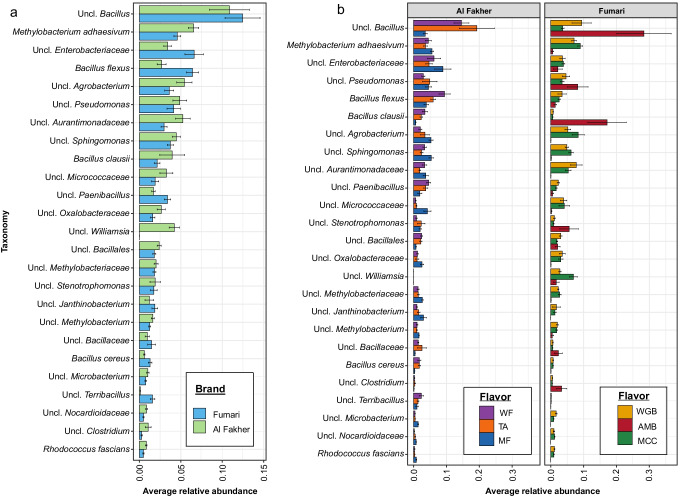
Average relative abundance of the top 25 bacterial taxa in **a** two brands of hookah tobacco products (Al Fakher and Fumari) and **b** different flavors of the two brands of hookah tobacco products (Al Fakher, watermelon (WF), two apple (TA), and mint (MF); Fumari, white gummy bear (WGB), ambrosia (AMB), and mint chocolate chill (MCC))

Regarding taxon composition of hookah-associated bacteria, the predominant bacterial taxa observed irrespective of brands and flavors were *Bacillus*, *Methylobacterium*, *Enterobacteriaceae*, *Agrobacterium*, *Pseudomonas*, *Aurantimonadaceae*, *Sphinogomonas*, *Micrococcaceae*, and *Paenibacillus* (Fig. 2a and b)*.* Among *Bacillus* species, we observed that *B. flexus* and *B. clausii* were significantly (*p* < 0.05 using DeSeq2) different between Al Fakher and Fumari brands, with *B. flexus* (6.4% + / − 0.007) more predominant in Al Fakher products while *B. clausii* (3.96% + / − 0.01) predominated in Fumari products (Fig. 2a).

A higher relative abundance of *Bacillus* genera (28.33% + / − 0.08) and *B. clausii* (17% ± 0.06) was observed in the Ambrosia flavor when compared to the other flavors within the Fumari brand. The predominant bacteria were *Bacillus* within the two apple (19.25% + / − 0.05) and the watermelon flavor (14.55% + / − 0.09) and the *Enterobacteriaceae* family (8.91% + / − 0.02) in the mint flavor. Additionally, we observed that the *Agrobacterium*, *Sphinogomonas*, *Paenibacillus*, and *Aurantimonadaceae* families were at a relative abundance less than 0.01% in the ambrosia flavor compared to all of the other hookah flavors. Overall, hookah tobacco products are host to diverse bacterial communities that differ significantly by brand and flavor.

### Effect of duration of storage on the microbiota of the brands and flavors

Storage of hookah under the three temperature and relative humidity conditions for 14 days did not significantly affect alpha diversity (observed richness and Shannon diversity; Supplemental Fig S4) (*p* > 0.05) and beta diversity (ANOSIM R =  − 0.0039, *p* = 0.992) (Supplemental Fig. S5) measures of hookah-associated microbiota. Therefore, the three storage conditions for a given timepoint were combined, and we considered them as biological replicates for further downstream analyses. Alpha diversity (observed species and Shannon diversity metrics) was characterized in samples from the two brands and flavors and compared between day 0 and day 14 (Supplemental Fig S6). Samples from the Al Fakher brand showed a significantly (*p* < 0.0001) decreased alpha diversity at day 14 compared to day 0, irrespective of flavors, for the Shannon diversity metric (MF, Shannon: 1.19 + / − 0.39; TA, Shannon: 0.78 + / − 0.26; and WF, Shannon: 0.66 + / − 0.29) but not for the Observed species metric. In the Fumari products, the mint chocolate chill flavor showed a significantly decreased number of observed species at day 14, while the white gummy bear flavor showed a significantly increased number of observed species and no significant changes in the Shannon diversity metric at day 14.

Comparisons of bacterial community structure using beta diversity analyses of Bray–Curtis dissimilarity showed that the days of incubation among the two brands had a significant effect (*p* < 0.05) on the bacterial community composition. Within flavors, we observed a distinct difference (Fig. 1d and e). Comparisons of days of incubation (ANOSIM R, 0.2926; *p* = 0.001) within the Al Fakher brand showed 14.4% variance between bacterial communities along the first principal component axis (axis 1) and 7.7% along the second principal component axis (axis 2). Overall, the diversity measures indicate that the bacterial microbiotas in hookah tobacco products are significantly influenced by days of incubation.

**Fig. 3:**
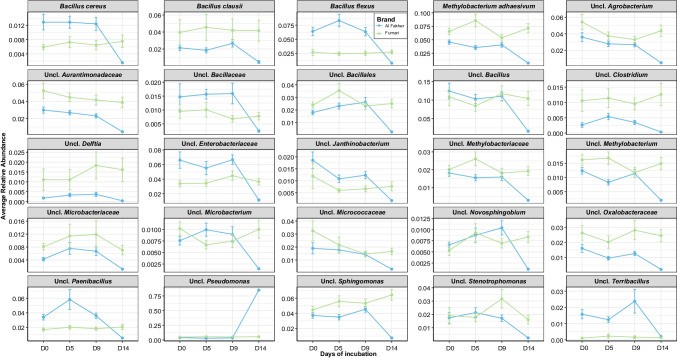
Longitudinal shifts in the average relative abundance (+ / − standard deviation) of the top 25 bacterial taxa over time (from D0 to D14) of incubation for Al Fakher (light blue) and Fumari (light green) brands

We also observed compositional differences between hookah brands through the 14-day incubation period (Fig. 3). In the Al Fakher brand, we observed a decrease in the relative abundance of most bacterial taxa (Fig. 3) except for *Pseudomonas* over time (Fig. 3). While in the Fumari brand, an increase in the relative abundance of certain bacterial taxa like *Delftia*, *Sphingomonas*, *Nocardioidaceae*, and *Clostridium* at day 14 was observed (Fig. 3 and S7). The same trend was generally observed when considering incubation time points 0 and 14 (Supplemental Fig. S7). These results clearly demonstrate that unlike chemical composition, which tends to be constant over time in tobacco products, bacteria associated with the hookah tobacco environment represents a dynamic system.

### Unique and shared bacterial taxa across hookah brands and flavors

**Fig. 4:**
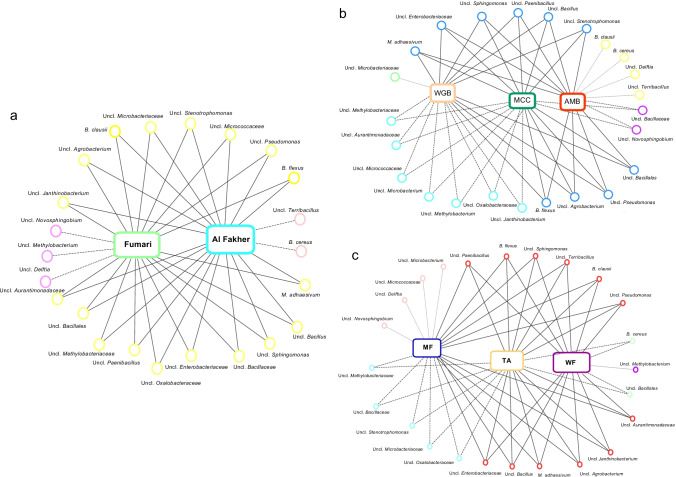
Bacterial profiles of shared and unique taxa between Hookah brands (**A**), Fumari flavors (**B**), and Al Fakher flavors (**C**) visualized by network plots in Cytoscape

Among the top 25 bacterial taxa, we observed three bacterial taxa that were unique to the Fumari brand (*Novosphingobium*, *Methylobacterium*, and *Delftia*) and two that were unique to the Al Fakher brand (*Terribacillus* and *Bacillus cereus*). The bacterial taxa that were shared between the two hookah brands were *Aurantimonadaceae*, *Bacillales*, *Methylobacteriaceae*, *Paenibacillus*, *Oxalobacteraceae*, *Enterobacteriaceae*, *Bacillaceae*, *Sphinogomonas*, *Bacillus*, *Methylobacterium adhaesivum*, *Pseudomonas*, *Bacillus flexus*, *Bacillus clausii*, *Agrobacterium*, *Janthinobacterium*, *Microbacteriaceae*, *Stenotrophomonas*, and *Micrococcaceae* (Fig. 4a).

In the Fumari brand, *Microbacteriaceae* was unique to the white gummi bear flavor, and *Bacillus cereus*, *Bacillus clausii*, *Delftia*, and *Terribacillus* were unique to the ambrosia flavor, while mint chocolate chill had no unique bacterial taxa. Shared bacterial taxa between mint chocolate chill and ambrosia were *Bacillaceae* and *Novosphingobium*, while *Aurantimonadaceae*, *Methylobacteriaceae*, *Micrococcaceae*, *Microbacterium*, *Methylobacterium*, *Oxalobacteriaceae*, and *Janthinobacterium* were shared between white gummi bear and mint chocolate chill. Bacterial taxa shared among the three hookah tobacco flavors were *Bacillales*, *Paenibacillus*, *Enterobacteriaceae*, *Sphinogomonas*, *Bacillus*, *Methylobacterium adhaesivum*, *Pseudomonas, Bacillus flexus*, *Agrobacterium*, and *Stenotrophomonas* (Fig. 4b)*.*

In Al Fakher hookah tobacco, four unique bacterial taxa were observed only in the mint flavor (*Novosphingobium*, *Delftia*, *Micrococcaceae*, and *Microbacterium*). Shared bacterial taxa between the mint flavor and the two apples flavor were *Methylobacteriaceae*, *Bacillaceae*, *Stenotrophomonas*, *Microbacteriaceae*, and *Oxalobacteriaceae* and between the two apple and the watermelon flavor were *Bacillales* and *Bacillus cereus*. Additionally, *Paenibacillus*, *Bacillus flexus*, *Sphingomonas*, *Terribacillus*, *Bacillus clausii*, *Pseudomonas*, *Aurantimonadaceae*, *Janthinobacterium*, *Agrobacterium*, *Methylobacterium adhaesivum*, *Bacillus*, and *Enterobacteriaceae* were shared between the three Al Fakher flavors (Fig. 4c). These results strongly corroborate with our diversity and compositional observations, indicating that the hookah microbiome strongly differs between brands and flavors.

## Discussion

It is well established that smokers are more susceptible to bacterial infections compared to nonsmokers (Bagaitkar et al. 2008; Feldman and Anderson 2013). In the case of hookah users, studies have shown that sharing the mouthpiece during a smoking session can transfer a wide range of pathogens, such as viruses, bacteria, and fungi, among users (Urkin et al. 2006; Martin et al. 2013; Balaky et al. 2018). Therefore, characterizing this exposure route as well as investigating the bacteria associated with hookah tobaccos is essential for understanding how hookah tobacco use impacts health. Our study not only demonstrates the presence of distinct bacterial communities in different hookah tobacco brands but also shows that the use of flavors and days of incubation can alter the bacterial community composition of the brands. However, we did not observe significant changes in bacterial community diversity when storage conditions were altered. This observation corroborates previous studies that investigated the effect of different storage conditions on the microbial diversity of commercial cigarettes (Chopyk et al. 2017b), little cigars (Smyth et al. 2019), and research cigarettes (Chattopadhyay et al. 2021). Previous studies have also shown that, unlike other tobacco products, hookah products contain higher levels of humectants like glycerol and honey (Khater et al. 2008; Uebelacker et al. 2019), both of which also exhibit bacteriostatic and bactericidal activities, which could help explain the lack of effect of incubation temperatures or humidity levels on hookah tobacco alpha-diversity.

The existence of appealing flavors, which are added to mask the harshness and discomforts of hookah smoking, are the primary reasons for the popularity of hookah use, particularly among young adults and women. To deter smoking in the USA, particularly among young adults and adolescents, a ban on flavored tobacco (except menthol) was imposed by the US Food and Drug Administration (FDA) under the 2009 Family Smoking Prevention and Tobacco Control act (US FDA 2020a). Recently, the FDA also banned mint- and fruit-flavored vaping products, exempting menthol and tobacco flavors (Lovelace Jr 2020).

Despite the misperception of hookah being less harmful than cigarette use, several studies report a potential risk for transmission of communicable diseases due to the unhygienic conditions of hookah apparatus and the sharing of mouthpieces (Martinasek et al. 2018; Akl et al. 2010; Szyper-Kravitz et al. 2001; Munckhof et al. 2003). For example, a survey of 15 restaurants and waterpipe cafes in Kerman city, Iran, revealed the presence of Coagulase-negative staphylococci, *Streptococcus*, *Staphylococcus aureus*, *Klebsiella*, *Neisseria*, *Pseudomonas aeruginosa*, *Bacillus*, and *E. coli* in the different components of the waterpipe (Safizadeh et al. 2014). Additionally, several studies in recent years have shown the presence of diverse microbial communities in several commercially available tobacco products. For instance, commercial cigarettes are known to harbor rich and diverse bacterial populations ranging from common soil microbes to potential human pathogens (Kurup et al. 1983; Rooney et al. 2005; Sapkota et al. 2009; Larsson et al. 2008; Eaton et al. 1995; Chopyk et al. and Malayil et al.). Similarly, studies from our lab and other research groups have shown the presence of diverse bacterial communities in little cigars and smokeless tobacco products (Tyx et al. 2016; Tyx et al. 2016; Chattopadhyay et al. 2019; Smyth et al. 2019). Nevertheless, the presence of microbes in hookah studies has been limited to those focused on the apparatus, leaving their characterization in hookah tobacco largely unexplored.

In this study, we observed a high relative abundance of *Pseudomonas*, *Bacillus*, *Methylobacterium*, and *Sphinogomonas* in all of the sampled hookah tobacco brands irrespective of flavors. Previously, Hani et al. (2018) demonstrated the presence of these bacteria in the physical components of the hookah apparatus, water, and the hookah tobacco. Although most of these bacteria are ubiquitous in the environment, some species within each of these genera can be opportunistic pathogens. In our study, we observed the presence of *B. cereus* and *S. multivorum* in Fumari products (Figs. 2, 4 and Supplementary Table S1), which have been associated with respiratory tract infections (Rooney et al. 2005; Lambiase et al. 2009). Recently, a 66-year-old male (frequent smoker) was diagnosed with a rare case of pleural infection caused by *Propionibacterium acnes* (skin commensal) (Cobo et al. 2018), a bacterial species we identified in our study in Fumari hookah products (Supplementary Table S1). Our study also observed the presence of *Paenibacillus lautus* in the Fumari products, which has not only been reported as an opportunistic pathogen, but is also resistant to many commonly used antibiotics (Loong et al. 2018) (Supplementary Table S1). In addition to human opportunistic pathogens, we also observed the presence of the phytopathogens *R. fascians* (leafy gall disease in tobacco) (Stes et al. 2013) (Fig. 2 and Supplementary Table S1) and *P. viridiflava* (bacterial blight) (Sarris et al. 2012) in Al Fakher tobacco products (Supplementary Table S1). The presence of these natural, commensal, and antibiotic-resistant bacteria in hookah tobacco could be of concern to the health of hookah shisha smokers.

Beyond human and plant bacterial pathogens, we also observed bacterial species that can tolerate heavy metals such as arsenic and degrade polycyclic hydrocarbons, such as nicotine and toluene, which are present in mainstream smoke. Similar bacterial genera were also previously observed in commercial cigarettes studied by our group (Malayil et al. 2020). For example, *B. flexus* and *P. veronii*, which were observed in both hookah brands (Figs. 2 and 3; Supplementary Table S1), are known to tolerate arsenic (Jebeli et al. 2017) and degrade toluene, both of which are found in mainstream smoke (Moldoveanu et al. 2008; Lazarević et al. 2012). Additionally, previous studies have isolated bacteria (*V. paradoxu*s, *Sphingomonas*, *Acinetobacter*, and *Pseudomonas*) that are capable of degrading nicotine (Ruan and Min 2005; Wang et al. 2011). Hence, it is critical to understand the role that these bacteria may play in the biotransformation of nicotine and toluene, thereby potentially reducing tobacco-induced damage among hookah tobacco users. Previous studies have shown that hookah smoke condensate contains harmful polycyclic aromatic hydrocarbons (PAHs), carbon monoxide and heavy metals (Qasim et al. 2019). Recently, Markowicz et al. (2014) have also identified the presence of LPS and fungal biomass in water pipe tobacco and smoke (Markowicz et al. 2014).

Strengths of this study include our analysis of several time points, and the direct comparison of two popular hookah brands, across multiple flavors. Study limitations include inherent biases introduced during PCR amplification, the absence of species-level assignments in some cases, and our inability to discern live from relic/dead bacterial communities within the hookah products.

Nevertheless, our study provides a comprehensive characterization of hookah bacterial communities and demonstrates that flavors and brands may alter the bacterial community composition, potentially selecting for bacteria including *Pseudomonas*, *Bacillus*, *Methylobacterium*, and *Sphingomonas* in certain brands. Therefore, hookah users’ exposures to bacterial constituents originating from hookah tobacco may be impacted differentially based on the users’ specific brand and flavor of choice.

## Supplementary Information

Below is the link to the electronic supplementary material.

Supplementary file1 (XLS 1578 KB)

Supplementary file2 (PDF 795 KB)

## Data Availability

Data concerning the samples included in this study are deposited in the NCBI BioProject database under BioProject accession number PRJNA641233.
