# Supplementary material for: Bacterial communities of hookah tobacco products are diverse and differ across brands and flavors
Source: Appl Microbiol Biotechnol. 2022 Aug 5;106(17):5785–95. doi: 10.1007/s00253-022-12079-7 (PMC9361917; doi:10.1007/s00253-022-12079-7)

Applied Microbiology and Biotechnology

Bacterial Communities of Hookah Tobacco Products are Diverse and Differ Across Brands and Flavors

Leena Malayil[1], Suhana Chattopadhyay[1], Emmanuel F. Mongodin[2&‡], Amy R. Sapkota[1‡*]

[1] Maryland Institute for Applied Environmental Health, University of Maryland School of Public Health, College Park, MD, USA

[2] Institute for Genome Sciences, University of Maryland School of Medicine, Baltimore, MD, USA. Current address: National Institutes of Health (NIH), National Heart, Lung and Blood Institute (NHLBI), Division of Lung Diseases, Bethesda, MD

[‡] Equal contribution, shared senior co-authorship

*Corresponding Author: Amy R. Sapkota, Ph.D., M.P.H. email: ars@umd.edu

[&] Current address: National Institutes of Health (NIH), National Heart, Lung and Blood Institute (NHLBI), Division of Lung Diseases, Bethesda, MD

Disclaimer: Dr. Mongodin contributed to this article as an employee of the University of Maryland School of Medicine. The views expressed are his own and do not necessarily represent the views of the National Institutes of Health or the United States Government

Figure S1: Boxplots displaying the number of 16S sequences per groups of hookah flavor samples after sequencing quality filtering. Bars are colored by flavors: Al Fakher (flavors: dark blue- Mint (MF), orange- Two apple (TA) and purple- Watermelon (WF)) and Fumari (flavors: red- Ambrosia (AMB), green- Mint chocolate chill (MCC) and brown- White gummy bear (WGB)).

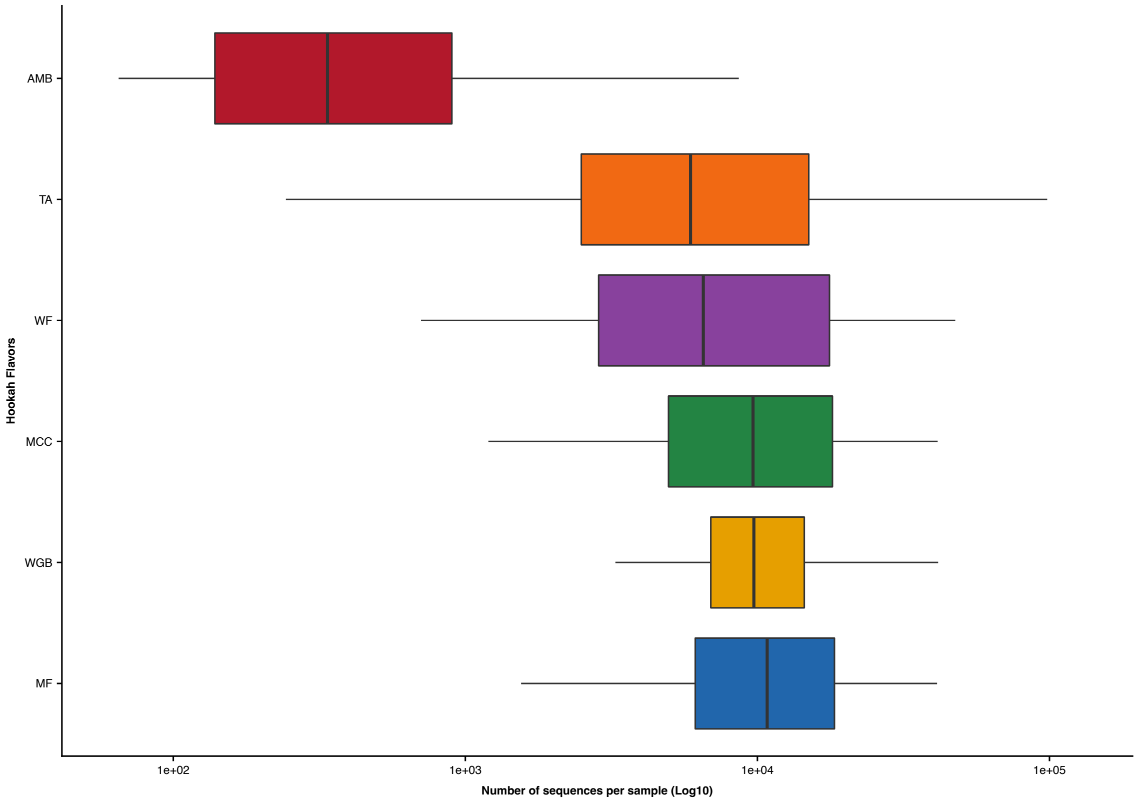

Figure S2: Good's estimate of sequence coverage across samples from the two hookah brands.

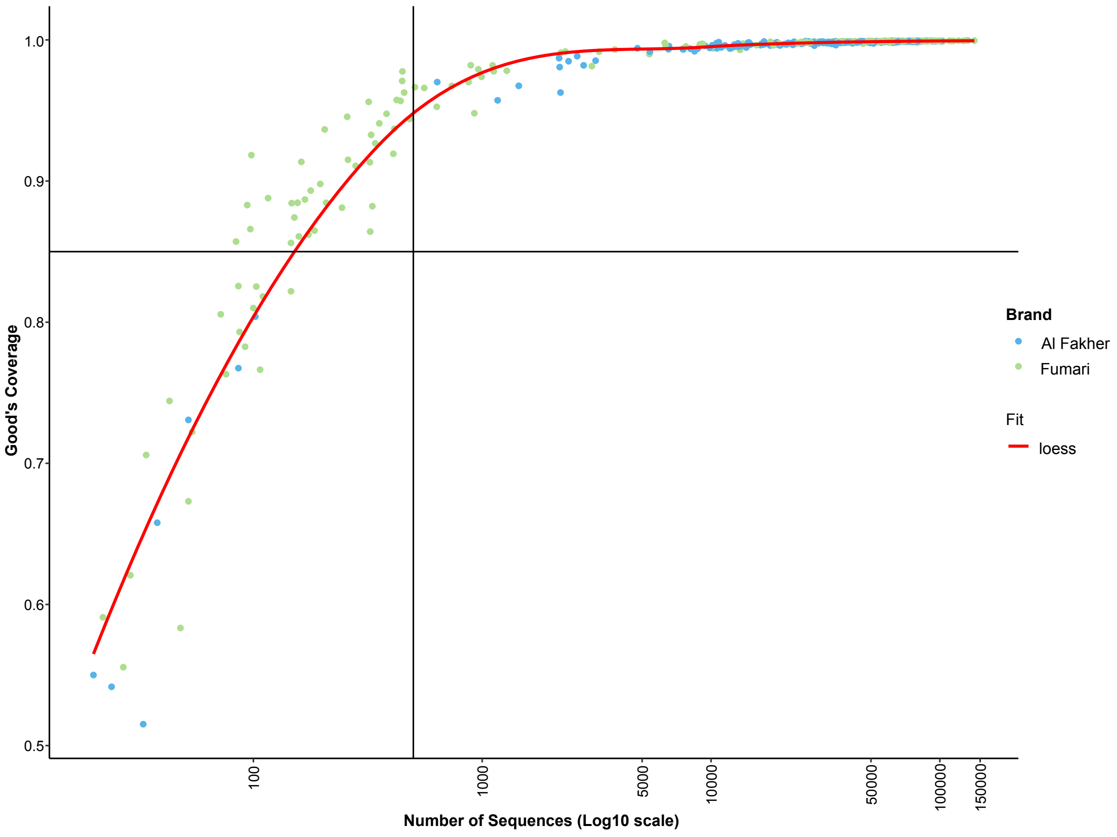

Figure S3: Violin box plots showing alpha diversity metrics (Observed number of species and Shannon Index) across samples on non-rarefied data, colored by flavors: Al Fakher (flavors: dark blue- Mint (MF), orange- Two apple (TA) and purple- Watermelon (WF)) and Fumari (flavors: red- Ambrosia (AMB), green- Mint chocolate chill (MCC) and brown- White gummy bear (WGB)).

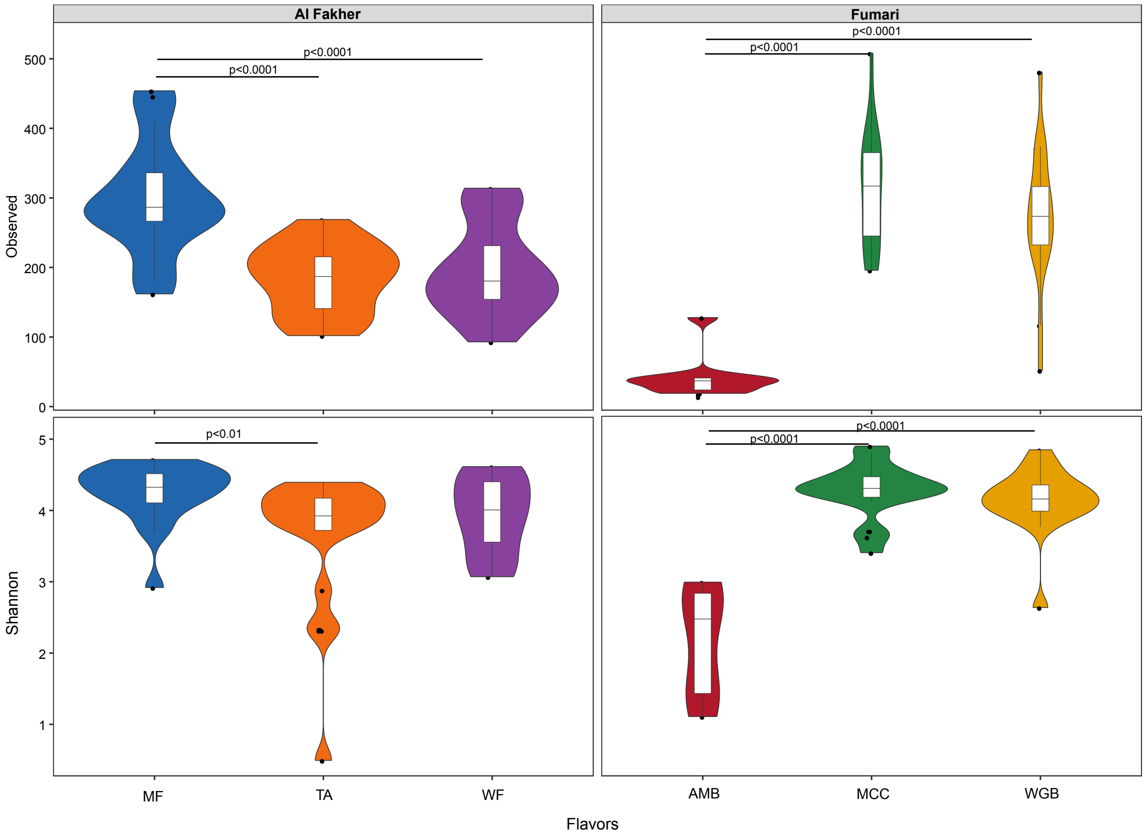

Figure S4: Violin box plots showing alpha diversity metrics (Observed number of species and Shannon Index) for samples from two hookah brands on non-rarefied data comparing three storage conditions for 14 days. Boxplots are colored by storage conditions: Fridge (green), Pocket (dark orange) and Room (maroon).

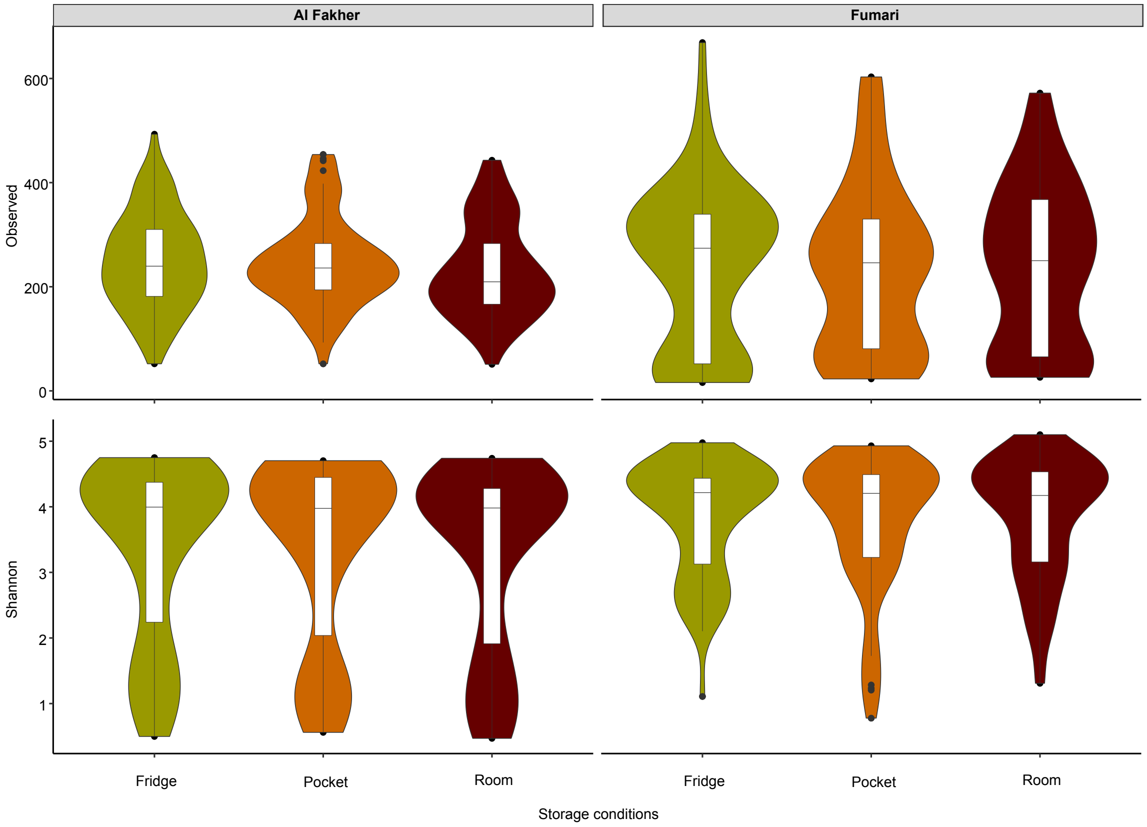

Figure S5: Principle coordinate analysis plots of Bray-Curtis computed distances between brand and storage condition at four different timepoints (Day 0, 5, 9 and 14). The colors represent the different storage conditions (Fridge (green), Pocket (dark orange) and Room (maroon)) and the shapes represents the brands: circle, Al Fakher; and triangle, Fumari. The dashed colored ellipses are drawn at 95% confidence intervals for storage conditions.

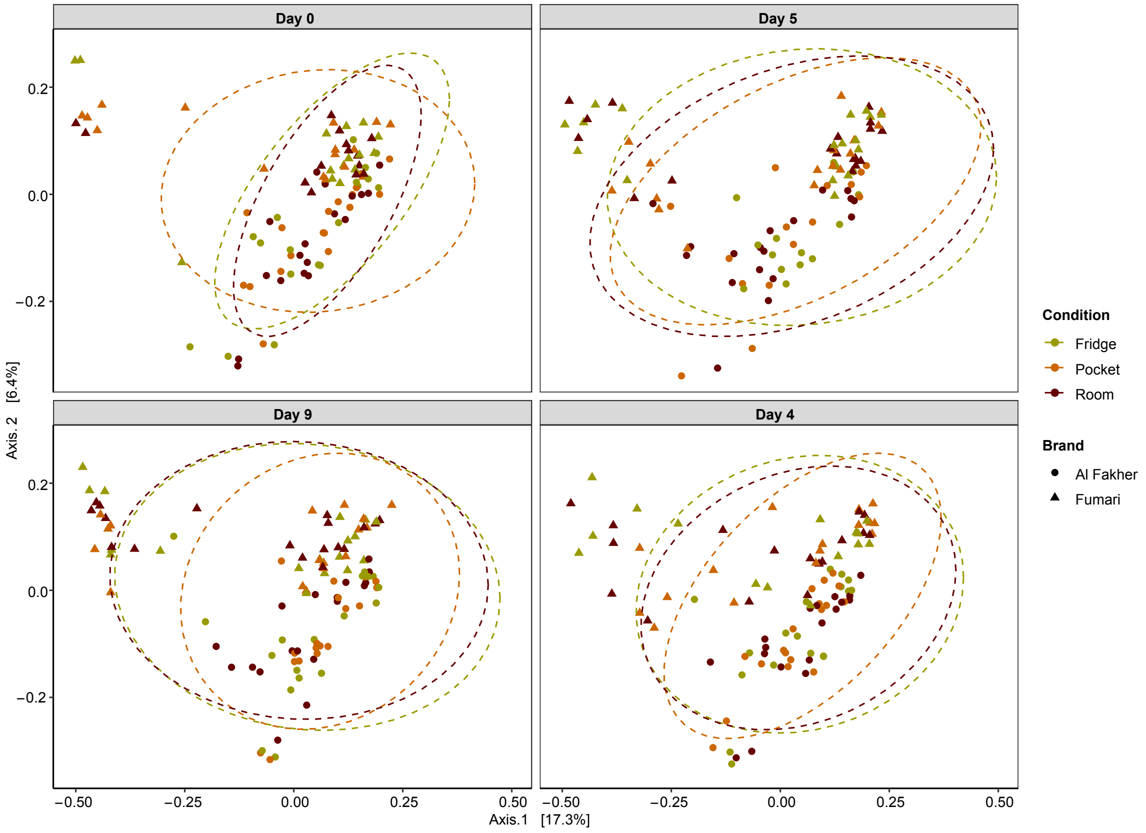

Figure S6: Violin box plots showing alpha diversity metrics (Observed number of species and Shannon Index) across samples on non-rarefied data comparing Day 0 to Day 14 timepoints. Boxplots are colored by flavors: Al Fakher (flavors: dark blue- Mint (MF), orange- Two apple (TA) and purple- Watermelon (WF)) and Fumari (flavors: red-Ambrosia (AMB), green- Mint chocolate chill (MCC) and brown- White gummy bear (WGB)).

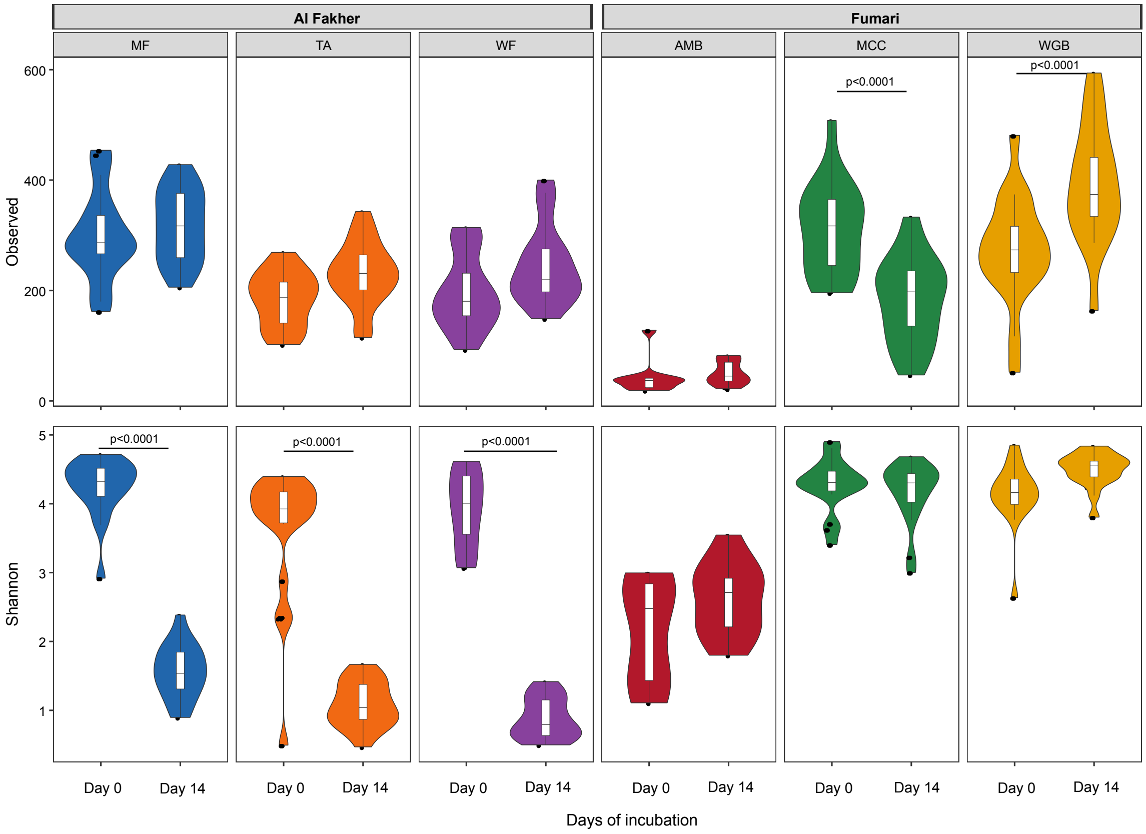

Figure S7: Line graphs of the average relative abundance (+/- standard deviation) of the top 25 bacterial taxa between Day 0 and Day 14 of incubation for Al Fakher (light blue) and Fumari (light green) brands.

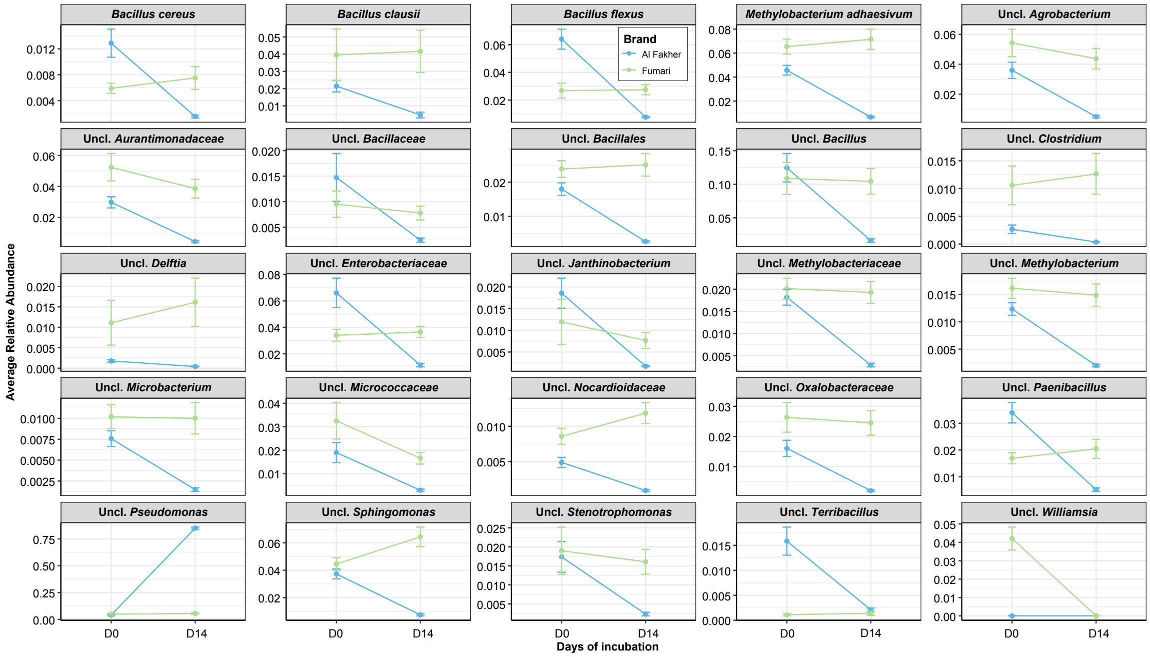

Supplement: Supplementary file 2 — Supplementary file2 (PDF 795 KB) [file 253_2022_12079_MOESM2_ESM.pdf]
